# Modern approach to the management of genitourinary syndrome in women with gynecological malignancies

**DOI:** 10.2478/raon-2023-0038

**Published:** 2023-07-26

**Authors:** Nina Kovacevic, Ines Cilensek, Sebastjan Merlo, Barbara Segedin

**Affiliations:** Department of Gynecological Oncology, Institute of Oncology Ljubljana, Ljubljana, Slovenia; Faculty of Medicine, University of Ljubljana, Ljubljana, Slovenia; Faculty of Health Care Angela Boškin, Jesenice, Slovenia; Institute of Histology and Embryology, Faculty of Medicine, University of Ljubljana, Ljubljana, Slovenia; Department of Radiotherapy, Institute of Oncology Ljubljana, Ljubljana, Slovenia

**Keywords:** genitourinary syndrome, gynecological malignancies, therapy

## Abstract

**Background:**

The term genitourinary syndrome of menopause was first used in 2014 by the North American Menopause Society and the International Society for the Study of Women's Sexual Health to describe conditions previously known as atrophic vaginitis, urogenital atrophy, or vulvovaginal atrophy. It is a complex, chronic, progressive condition characterized by a wide range of signs and symptoms affecting sexual function and the tissues of the urinary and genital tracts. The main cause of genitourinary syndrome of menopause is estrogen deficiency caused by ovarian removal or dysfunction. The most bothersome symptoms are vaginal dryness, decreased vaginal lubrication, and pain during penetration and intercourse. They all have a negative impact on the quality of life.

**Conclusions:**

The main goal of treatment is to relieve the symptoms. Treatment modalities are pharmacological or non-pharmacological. The first-line treatment for mild to moderate symptoms is the use of personal lubricants and moisturizers, but the gold standard is estrogen replacement therapy. Hormone therapy may not be an option for women with hormone-dependent cancer.

## Introduction

Gynecological malignancies account for approximately 10% of all cancers in women, and 40% of patients are premenopausal at the time of diagnosis.^1,2^ Treatment of gynecological malignancies is often multimodal with surgery (hysterectomy with bilateral salpingo-oophorectomy), systemic therapy, and radiation leading to induced menopause. This hypoestrogenic state can lead to menopausal symptoms and can negatively affect sexual quality of life.^3^

Genitourinary syndrome of menopause (GSM) is a new term that describes conditions formerly known as vulvovaginal atrophy, atrophic vaginitis, or urogenital atrophy, all of which result from estrogen deficiency.^4^ GSM is a chronic, progressive condition that causes multiple changes in the vulvar and vaginal area, pelvic floor tissues, bladder, and urethra, and impairs sexual function and libido. These changes occur in response to hypoestrogenism and do not improve with time.^5^ GSM affects 27 to 84% of menopausal women.^6^ Women treated for gynecological malignancy may enter menopause earlier than healthy women.

Many survivors of gynecologic malignancies experience GSM symptoms, which impair their quality of life. Compared to healthy controls, women after surgery for early cervical cancer and endometrial cancer report sexual desire dysfunction, arousal dysfunction, entry dyspareunia and reduced intensity orgasm more often.^7,8^ Fertility-sparing procedures may preserve childbearing potential, but they do not have the impact on sexual satisfaction.^9,10^

The addition of radiotherapy can additionally impair vaginal function, resulting in loss of elasticity and fibrosis of vaginal walls. After curative radiotherapy for cervical cancer, less women are sexually active compared to the time before treatment. Women report vaginal functioning problems, such as vaginal dryness, shortening and/or tightening of the vagina, which in turn correlate with diminished sexual enjoyment.^11^ 45% of patients are not capable of full intercourse after curative radiotherapy for cervical cancer.^10^

Given high prevalence of GSM, it is important for physicians to address this issue. Women are often hesitant to address sexual and vaginal health issues, which are still considered taboo and are relieved when physicians bring up the topic. Because GSM is a chronic, complex condition, lifelong treatment is required to prevent recurrence of symptoms.^12^

We must keep in mind that up to 18% of endometrial carcinomas occur in women younger than 40 years. A multidisciplinary approach should be taken whether bilateral salpingo-oophorectomy is required as part of the staging procedure and when we can avoid problems with menopause.^13^ Similarly, ovarian transposition should be recommended for cervical cancer in premenopausal patients undergoing pelvic irradiation to avoid premature menopause and menopausal symptoms.^14^

We will discuss treatment modalities for GSM in women with gynecological cancer, considering both hormonal and non-hormonal options.

## Assessment

The clinical manifestations of GSM can be mild and nonspecific, so diagnosis may prove difficult. A careful assessment and identification of the most bothersome symptoms and their impact on quality of life should be performed before choosing a therapeutic approach. Simple and effective questionnaires are available, including the Vulvovaginal Symptoms Questionnaire (VSQ), the Sexual Symptom Checklist for Women After Cancer and EORTC QLQ CX-24, to assess symptoms, emotions, impact on life, and sexuality.^15,16^ In addition, a complete medical and gynecological history and a gynecological examination are required. The examination should include inspection of the external genitalia, vaginal inspection with a speculum, and bimanual palpation to rule out other conditions that may mimic GMS, such as urinary tract infections, vulvovaginal infections, allergic reactions, and urinary incontinence.^5,17^

## Symptoms and clinical manifestation

Hypoestrogenism due to bilateral oophorectomy or ovarian failure and pelvic irradiation results in anatomic and functional changes in urogenital tissue. There is loss of collagen and elastin in the vaginal epithelium, smooth muscle function is altered, and the number of small blood vessels is reduced, resulting in local tissue hypoxia. The increase in connective tissue leads to decreased elasticity, thinning of the epithelium and weakening of the vaginal mucosa.^6,18,19^

GSM presents as a wide range of signs and symptoms, the most common are summarized in Table 1. Dyspareunia and vaginal bleeding due to vaginal dryness are the most common symptoms of GSM.^12^ Vaginal dryness affects up to 93% of women, and burning, itching, and pruritus affect up to 63% of women. The most common sexual complaints are decreased vaginal lubrication and dyspareunia, affecting 90% and 80% of women, respectively. Urinary symptoms, dysuria, and incontinence are less common, affecting 29% and 25% of women, respectively.^5,20^

**TABLE 1.** Genital, urinary, and sexual signs and symptoms of genitourinary syndrome of menopause

**Genital**	**Urinary**	**Sexual**
Vaginal dryness	Dysuria	Dyspareunia
Vaginal irritation	Urgency	Decreased lubrication
Vaginal burning	Frequency	Postcoital bleeding and spotting
Vaginal itching	Reccurent urinary tract infections	Decreased arousal
Vulvar pruritus	Cystocele	Dysorgasmia
Thinning and graying pubic hair	Stress urinary incontinence	Loss of libido
Vaginal/pelvic pain and pressure	Urge urinary incontinence	Loss of arousal
Vaginal vault prolapse	Hematuria	Pelvic pain
Vaginal and introital stenosis	Nocturia	
Palor of vaginal mucosa		
Fewer vaginal rugae		
Petechiae in vaginal and cervical mucosa		
Labial shrinking and atrophia		

## Treatment approach

The main goal of GSM management is to relieve symptoms. The approach varies depending on the severity of symptoms. For severe and moderate symptoms, pharmacological treatment with hormone therapy (HT) is the gold standard. For mild symptoms nonhormonal therapies are subjectively effective. Nonhormonal therapies may also be used if gynecologic cancer is responsive to estrogen.^6,12^ Available treatment modalities are listed in Table 2.

**TABLE 2.** Pharmacological and non-pharmacological treatment modalities for the genitourinary syndrome of menopause

**Pharmacological treatment**	**Non-pharmacological treatment**
Hormone therapy	Lifestyle changes
SERM	Vaginal lubricants
DHEA	Vaginal moisturizers
Testosteron	Laser therapy
Lidocain	Vaginal dilatators

DHEA = dehydroepiandosterone; SERM = selective estrogen receptor modulator

### Pharmacological treatment

#### Hormone therapy

HT is the most effective therapy for GSM, but it is underutilized in women with gynecologic cancer.^6^ Systemic HT is acceptable for early stage endometrial cancer (FIGO stage I–II), but is not recommended for late stage endometrial cancer (FIGO stage III–IV). Systemic HT is also not recommended for uterine sarcomas, especially leiomyosarcomas and endometrial stromal sarcomas that express estrogen and progesterone receptors.^21^ According to the data, HT can be prescribed to women with epithelial ovarian cancer, but low-grade serous cancer may respond to anti-estrogen treatment, so systemic HT is not recommended in this ovarian cancer subtype. In clear cell carcinoma HT has generally been considered appropriate, but this hystological ovarian subtype itself has been associated with higher rate of venous thrombembolic events.^22^ HT is also safe and acceptable in women with cervical cancer.^2,13^ Recommendation for HT use in women with different gynecologic malignancy are shown in Table 3.

**TABLE 3.** Recommendations for hormone therapy in women treated for gynecological malignancies

**Gynecological malignancy**	**Recommendation**	**Selected articles**	**Level of evidence**	**Note**
**Uterine cancer**				

Early stage endometrial cancer	HT acceptable	Barakat *et al*. 2006^31^	randomized control trial	1236 patients, no difference in recurrence rate with the use of HT
		Shim *et al.* 2014^32^	meta-analysis	no increased risk of recurrence
Advanced stage endometrial cancer	HT not recommended	Sinno *et al.* 2020^2^	NAMS clinical practice statement	no data supporting use of HT
Uterine sarcoma	HT not recommended	George *et al.* 2014^21^	phase 2 trial	27 patients, a potential response to anti-estrogen therapy (Letrozole)
		Sinno *et al.* 2020^2^	NAMS clinical practice statement	lack of data regarding HT safety

**Ovarian cancer**				

High grade serous	HT acceptable	Li *et al.* 2015^33^	meta-analysis	HT is not associated with poorer clinical outcome, epithelial ovarian cancers
Low grade serous	HT not recommended	Gershenson *et al*. 2012^34^	retrospective study	64 patients, high rate of hormone receptor expression and maintenance anti-endocrine therapy
		Sinno *et al*. 2020^2^	NAMS clinical practice statement	not sufficient safety data available
Endometrioid	HT acceptable	Power *et al*. 2016^35^	retrospective cohort data	391 patients, HT is not associated with decreased disease-free or overall survival
Clear cell	HT not recommended	Didar *et al*. 2023^22^	meta-analysis	increased risk of venous thromboembolism events
Mucinous	HT acceptable	Li *et al*. 2015^33^	meta-analysis	HT is not associated with poorer clinical outcome, epithelial ovarian cancers

**Cervical cancer**	HT acceptable	Ploch *et al.* 1987^36^	prospective study	120 patients, no difference in recurrence rate with the use of HT

HT = hormone therapy; NAMS = North American Menopause Society

In women with moderate to severe GSM symptoms, the use of local estrogen might be suggested. Up to 45% of women find systemic HT insufficient to control GSM symptoms, whereas local HT is highly effective and provides symptomatic relief. The lowest dose for the shortest duration appropriate to treatment goals should be used. Estrogens and progestogens are the main hormonal preparations used in HT. Although both classes of hormones may have symptomatic benefits, progestogen is specifically added to estrogen regimens, unless the uterus has been removed to avoid endometrial hyperplasia and the increased risk of endometrial cancer. Premenopausal patients treated with curative radiotherapy with a dose of 80 Gy or more, may have symptoms of residual functional endometrium and should be advised to use estrogens in combination with a progestogen, instead of unopposed estrogens, to prevent stimulation of residual functional endometrium.^23,24^

HT is available through a variety of different routes of administration.^25^ Estrogen can be administered either locally or systemically. Systemic oral, transdermal (patch and spray), intranasal, sublingual, buccal, vaginal, subcutaneous, and intramuscular routes of administration of estrogen are possible, as are oral, vaginal, transdermal, intranasal, buccal, intramuscular, and intrauterine applications of progestogens.^26^

Vaginal estrogen is administered locally as a cream, gel, ring, or vaginal tablet, with minimal systemic absorption.^27,28^ With vaginal application of 10 mcg of estrogen, systemic estrogen concentrations remain in the postmenopausal range.^29^ There is no good evidence to support the use of a specific local estrogen product. In a retrospective cohort study of 244 women, treated for cervical, endometrial or ovarian cancer, symptom improvement was documented in one third of patients, unfortunately data on treatment efficacy is lacking for almost 60% of patients. With an incidence of 7.1%, 21.7%, and 9.7% of combined local and systemic recurrences in endometrial, ovarian, and cervical cancer, respectively, and a low incidence of other adverse outcomes, treatment was considered safe.^30^ Local vaginal estrogen therapy may be considered in women with hormone-dependent cancer if symptoms persist and nonhormonal treatment has failed. This should be an informed shared decision between physician and patient.^17,27^

In a randomized double-blind trial of 1236 women surgically treated for early-stage endometrial cancer and treated with estrogen replacement therapy versus placebo for GSM, 70.1% of estrogen-treated women experienced improvement in symptoms. The recurrence rate was low at 2.1%.^31^

In a prospective cohort study of 1045 patients treated for locally advanced cervical cancer with chemoradiotherapy and brachytherapy, hormone replacement therapy was associated with less vaginal dryness (28% *vs*. 18%), less vaginal shortening (27% *vs*. 17%), and less pain during intercourse (23% *vs*. 12%).^11^

#### Selective estrogen receptor modulator

Ospemifene is a selective estrogen receptor modulator (SERM) and is approved for the treatment of moderate to severe dyspareunia, a symptom of vulvovaginal atrophy. It acts as an estrogen agonist on vaginal tissue and the endometrium, with no systemic effects on bone, breast, or cardiovascular health.^37^ A meta-analysis conducted by Cui *et al*. showed that daily use of 60 mg ospemifene per os improved vaginal structure in terms of decrease in vaginal parabasal cells, increase in superficial vaginal cells, and decrease in vaginal pH. The differences in endometrial thickness at weeks 12 and 52 were significant and reflected greater thickening associated with ospemifene. Endometrial thickness was also assessed, and biopsies did not show endometrial hyperplasia or carcinoma with either short- or long-term use.^38,39,40^ However, ospemifene is not recommended for estrogen-dependent malignancies.^17^

#### Dehydroepiandosterone

Dehydroepiandosterone (DHEA) is a source of sex steroid hormones produced by the adrenal gland, and it is useful in treatment of vaginal dryness and dyspareunia. DHEA is metabolized to estrogens in vaginal mucosal cells and improves symptoms of vaginal irritation.^28^ Studies showed that DHEA administered intravaginally for 12 weeks improves vaginal cytological environment, lowers vaginal pH, and promotes cell maturation, resulting in symptom relief. Vaginal DHEA affects serum androgen and estradiol concentrations, which increase as a result, making the safety of DHEA use in hormone-dependent cancers an issue.^27,41,42^

#### Testosteron

Vaginal tissue is rich in testosterone receptors, so intravaginal testosterone is sometimes used off-label for GSM treatment. The enzyme aromatase converts testosterone to estradiol, so there are legitimate concerns about the safety of elevated serum estradiol levels in response to testosterone treatment in patients with hormone-dependent cancers.^17,27^ To date, hypoactive sexual desire disorder is the only evidence-based indication for the use of testosterone in postmenopausal women.^42^

#### Lidocain

If women suffer from penetrational dyspareunia, topical lidocaine can be used on the vaginal vestibule. In a randomized study, women who applied liquid lidocaine to the vaginal vestibule 3 minutes before intercourse reported less pain during intercourse and more comfortable penetration compared with the use of saline.^43^

### Nonpharmacological treatment

#### Vaginal lubricants and moisturizers

Lubricants and moisturizers should be used as first-line treatment for immediate discomfort and pain relive during intercourse, especially in women with hormone-dependent cancers. Lubricants are water-, oil-, mineral oil-, plant- or silicone-based and are not absorbed by the vaginal mucosa.^27^ They are applied before intercourse and have a temporary effect to reduce vaginal wall friction and relieve pain and discomfort during penetration and intercourse.^18^ Moisturizers are used regularly, from daily application to once every 2–3 days. They lower vaginal pH and hydrate vaginal mucosa. They alter vaginal epithelium by absorbing and adhering to it and mimicking vaginal secretion. The effect lasts up to a few days.^5^ Moisturizers are also recommended for women who are not sexually active and experience symptoms of vaginal dryness. There is a wide variety of over-the-counter lubricants and moisturizers, but women should be counseled regarding pH and osmolarity. The WHO recommends an osmolarity of no more than 380 mOsm/kg to avoid damage to the vaginal epithelium. However, most commercially available products exceed this value, so an osmolarity of up to 1200 mOsm/kg is generally accepted. In healthy women, normal vaginal pH is between 3.8 and 4.5, and lubricants or moisturizers should adhere to this range and not have a pH below 3. Additives such as parabens, microbicides and glycols should also be avoided, because they can irritate the vaginal tissue and mucosa.^44^ The main limitation of using lubricants and moisturizers is short-term relief of symptoms and the fact that they do not reverse atrophy. They are suitable for mild to moderate GSM symptoms and daily wellbeing.^3^ Women should be advised on which products are suitable, to avoid further damage to the vaginal epithelium.

#### Lifestyle changes

With regard to a conservative approach, smoking cessation is recommended as one of the GSM treatment modalities. Cigarette smoking has a negative effect on the vaginal epithelium, leading to a lack of vaginal cell maturation and increasing vaginal cell atrophy.^45^ Regular sexual activity with or without a partner is recommended to maintain vaginal elasticity, blood circulation, and lubrication during arousal.^18^ Regular exercise for pelvic muscle strengthening and relaxation are also advised. If available, psychosexual support should be offered.^10^ Consumption of nutrients containing equol, which is produced by equol-producing bacteria from isoflavonoids, showed a beneficial effect on alleviating vaginal symptoms in GSM.^46,47^

#### Laser therapy

In the last 5 years, laser use has gained popularity and has become an innovative treatment method for GSM. It is used as a minimally invasive technique that generates pulses that act on the vaginal mucosa. Epithelial cellularity and proliferation are increased, resulting in neoangiogenesis and neocolagenesis in the lamina propria of the vaginal mucosa.^48^ When using lasers for GSM treatment, microablative CO_2_ lasers or nonablative vaginal erbium Yag lasers are an option.^17^ The most common energy setting for CO_2_ lasers is 30–40 W and 3–10 J/cm^2^ for erbium Yag lasers. In a phase I–II study, progressive increase in vaginal length and improvement in vaginal health index was achieved with laser treatment, however, this did not transfer into improvement of female sexual function index.^49^ The efficacy of laser treatment for GSM caused by hormone therapy for breast cancer and in general population of postmenopausal women has been demonstrated in several retrospective series.^48^

In general, laser treatment appears to be safe and effective for GSM treatment, and no serious adverse events have been reported.^48^ In women who prefer nonhormonal treatment, laser treatment may be considered, but they need to be informed about the lack of data on long-term safety and efficacy of various laser therapies for GSM symptoms.^12,17,48^ In Slovenia laser treatment is not reimbursed by health insurance.

#### Vaginal dilators

Due to surgery and/or radiation therapy, the elasticity or length of the vagina may be compromised. In such cases, vaginal dilators can be helpful. In the early stages, dilators prevent or minimize the formation of adhesions between vaginal walls and promote elasticity and reduce fibrosis in later stages.^37^ The use of vaginal dilators should begin no later than 3 months after the end of radiotherapy and should be performed at least 2 to 3 times per week for 10 to 15 minutes to achieve positive effects on vaginal stenosis.^50^ It is important to educate women on how to relax the pelvic muscles and provide them with guidelines and instructions on dilators and their use.^19^ In a randomized trial the women who regularly used vaginal dilators after radiotherapy had less frequent and less severe vaginal stenosis.^51^

## Conclusions

Anatomic, physiologic, and sexual changes after treatment of gynecological malignancies are common and negatively impact quality of life and recovery from cancer. Physicians need to be aware of underestimated GSM symptoms and manifestations and address this issue with their patients. The treatment modality of GSM should be evaluated on an individual basis. The first-line treatment is non-homone approach, but if this fails, the use of local estrogen therapy could be used, taking into account the subtype of gynecologic malignancy.
